# Macular serpiginous choroiditis – case report

**Published:** 2018

**Authors:** Roxana Cozubas, Emil Ungureanu, Sinziana Luminita Instrate, Cristina Alexandrescu, Razvan Vladimir Nanu, Laura Carstocea, Liliana Mary Voinea, Radu Ciuluvica

**Affiliations:** * ”Grigore Alexandrescu”, Emergency Hospital for Children, Bucharest, Romania; ** Ophthalmology Department, University Emergency Hospital; “Carol Davila” University of Medicine and Pharmacy, Bucharest, Romania; *** “Sf. Ioan” Emergency Hospital, Bucharest, Romania; **** PhD Student, “Carol Davila” University of Medicine and Pharmacy, Bucharest, Romania; ***** Anatomy Department, “Carol Davila” University of Medicine and Pharmacy, Bucharest, Romania

**Keywords:** serpiginous choroiditis, white dot syndrome, uveitis

## Abstract

Serpiginous choroiditis represents an inflammation, often asymmetric, situated at the level of the inner choroid, which is related to the retinal pigment epithelium and the choriocapillaris. It is known as a primarily idiopathic, but there are authors who consider it an autoimmune process. Many times, fundus autofluorescence is the investigation which is guiding the diagnosis, the management, also the follow-up together with the determination of the progressive visual prognosis. Local treatment is proven to be sometimes an adjunctive treatment often effective.

**Abbreviations:** SC = Serpiginous choroiditis; RPE = Retinal pigment epithelium

## Introduction

SC is a term used for inflammatory disease, which typically extends from the retina in the peripapillary area. The geographic pattern also affects the overlying RPE and, in most of the cases, the outer retina [**1**]. When it is diagnosed, it usually involves the both eyes; it can also lead to irreversible damage, due to the localization, at the level of the photoreceptors; if it is situated at the level of the fovea, it will cause permanent vision loss [**2**]. 

Unfortunately, the specific trigger remains unknown, although there can be an underlying autoimmune process [**3**], incited by microorganisms – studies made with polymerase chain reaction (PCR) [**4**,**5**]. The response appears through an active proliferation, but many times can be because of an induction of the immune response which is made against the microbes. 

**Clinical features**

The typical manifestation consists of a choroiditis, which will extend in the retina, from the peripapillary area; the lesions have a grayish-yellow discoloration with a minimal, but also no inflammatory cell infiltration seen in the vitreous [**6**]. New lesions are usually symptomatic, and patients complain of blurred vision, metamorphopsia, paracentral scotomas, and floaters [**6**,**7**]. 

Visual acuity is 1/ 2 or less and slit lamp examination shows a quiet eye. Anterior chamber has a low grade of reaction, if present, with normal intraocular pressure [**8**]. The newer lesions are always well-circumscribed patches like grayish-white or with yellow discoloration found in the deep layers of the retina and RPE. In this area, retina and RPE which are outside the margins of an active or old lesion can appear normal. The fellow eye can be present with some similar atrophic lesions. 

In weeks or months, the grayish lesions will be replaced with a typical aspect of mottled RPE, together with a pigment epithelial hyperplasia and also a fibrosis. The active and also the healed lesions are, in this case, a strong evidence in sustaining the diagnosis [**9**]. The inactive lesions appear as a geographic atrophic area, with sharp borders and pseudopodia extensions [**10**]. 

The natural course is variable, with multiple recurrences of inflammation over years, and may involve the fovea [**10**]. If the treatment is not proceeded, the choroiditis will end up with an extensive scarring. Unfortunately, the disease is bilateral, but also asymmetrical, with a second eye involvement in about five years [**11**]. Macular involvement appears in 88% of the untreated patients [**12**]. 

## Case report

**Fig. 1:**
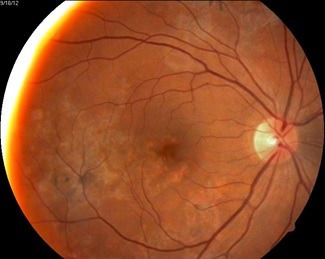
Right eye - macular involvement

About one third of the patients had the lesions initially or exclusively in the macula (**Fig. 1**). Our patient is a 43-year-old woman who is presenting with metamorphopsia, flashing lights, scotomas, a drop in vision in right eye and also floaters. Visual acuity at the time of presentation was 0.5 wc for the right eye; 1 wc for the left eye. 

**Fig. 2:**
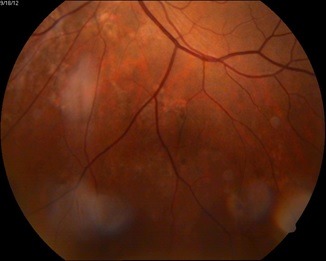
Left eye - lesions are more peripherally

At fundus examination, the right eye had macular implication (**Fig. 1**), whereas at the level of left eye had lesions more peripherally (**Fig. 2**). 

Diagnosis is usually challenging and that is because the new lesions will lack the atrophic scars, but also because the area in the peripapillary choroid could not be involved. If macular lesions are involved it can be better to proceed to extensive investigations for us to rule out infection etiology. Differential clinical diagnosis is made with neovascular membranes (subretinal), age related macular degeneration, retinal pigment epitheliitis and persistent placoid maculopathy [**13**]. 

Visual prognosis is poor, because of the neovascularization in choroid, which develops more frequently – 3 out of 7 patients [**13**]. Usually, the patients will be put to a more prompt treatment since the drop in vision is significant; this thing will result in a less extensive involvement at the level of the retina, but also in choroid layers [**14**]. 

Optical coherence tomography (OCT) will include a disruption of photoreceptor layer, which will bring out an outer retinal, bu also a choriocapillaris hyper-reflectivity [**15**]. The inner retina will be usually spared, but at the level of the outer layers of the retina we will find hyper-reflective appearance in the healed lesions, which are originated from the RPE proliferation, but also from its migration. The retinal inflammation in the active disease is here present with a minimal sub-retinal fluid which overlies to the area with the disease. When it comes to the OCT features, both active together with old lesions have similar appearance in OCT, but they have some differences regarding the retinal thickness, which can be normal in the active phase, but will be attenuated in atrophy phase [**16**]. 

The “waterfall effect” refers at the hyper-reflectivity found at the level of the choroid, on OCT, because here, the choroid has a cell infiltration [**17**]. In the old lesions, the hyper-reflectivity is enhanced by a light transmission at the level of RPE atrophied. Therefor the use of OCT is beneficial in providing details on development and extent of the complications – subretinal fluid, epiretinal membrane, cystoid macular edema, and subretinal neovascularization [**18**]. 

**Fig. 3:**
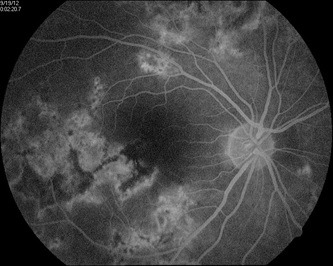
New lesions are slightly hypofluorescent

**Fig. 4:**
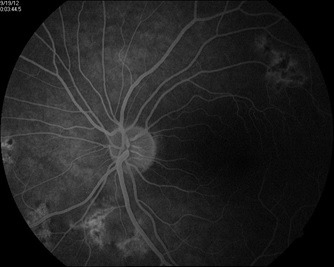
A halo appears at the level of the border of the active lesions

When it comes to the fundus auto-fluorescence, this imagistic investigation has features which are derived from accumulation of the lipofuscin made within the cells in RPE, thus revealing changes at the level of RPE layers. Here, the damage is detected early – we will find new lesions which are slightly hypo-fluorescent (**Fig. 3**,**4**), because of the masking effect or because the retina in outer layers is edematous. In two-five days, the area had become hyper-fluorescent, which is corresponding to the lipofuscin accumulated in RPE. A halo usually will appear at the level of the borders of the active lesion. This halo is originated from the thickening at the level of the cells in RPE, pushed by edema (**Fig. 3**). 

The hypofluorescent rim corresponds to the depigmentation at the level of RPE which is seen on direct ophthalmoscopy [**18**]. In weeks, the hyperfluorescence becomes speckled, until becoming undetectable [**18**]. The shift from a hyper to a hypo-fluorescence is made by the progressive atrophy and also the degeneration of the RPE, in cells level. 

The treatment may have influences in autofluorescence: in lesions which were treated in early stages with corticosteroids, we will observe a less hypo-fluorescence, when comparing those with the lesions not treated in the active stage; that is why the early anti-inflammatory proceeded treatment will reduce the damage made at the level of the RPE [**18**]. 

Visual fields defects are hardly indicated through the visual acuity, making perimetry an important asset for detecting an active stage of choroiditis and also the inactive atrophic areas of the retina. Dense scotomas from the central, but also from the peripheral lesions were documented with a Goldmann perimeter; Amsler grid test is also successful in this area [**19**].

From the visual field studies it is now confirmed that the layers situated in the inner retina are preserved (also nerve fiber layer included), whereas the layers in the outer retina is always destroyed [**20**]. 

## Differential diagnosis

Regarding the differential diagnosis in terms of SC is made with similar lesions, like infectious choroiditis (Mycobacterium tuberculosis, herpes viruses, syphilis). In these cases, lesions are multifocal, sparing the juxtapapillary area [**4**]. 

**Fig. 5:**
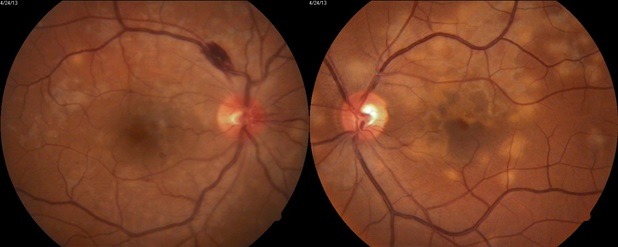
Acute posterior placoid pigment epitheliopathy

Another type of lesion, acute posterior multifocal placoid pigment epitheliopathy refers to an acute, self-limited, multifocal, and bilateral disease at the level of RPE, which resolves spontaneously [**21**]. Acute lesions may resemble SC (**Fig. 5**), but they resolve in 2 weeks, with the minimum RPE destruction. Visual prognosis is good, but also the condition is non–recurrent [**22**]. 

## Complications

In SC, the main threat is involvement of the fovea SC [**17**]. If the lesions appear in the fovea part of the retina or para-foveal, but also if the extra-foveal placoid lesions will complicate with neovascularization, then we will attend a central vision affection. Neovascularization is present in like 35% of the eyes that develop SC and appears usually in the healed placoid lesions, but also in the surrounding area [**16**].

Other complications are not constantly reported: retinal vasculitis, ischemia at the level of the retina, serous retinal detachment, vascular occlusions, together with secondary neovascularization, but also with retinal and vitreous hemorrhage [**16**,**17**]. 

## Treatment

When it comes to systemic treatment, this one is based on the pathogenesis which is presumed autoimmune for the SC. Thus, the immunosuppressive agents will be the best option in this case. While it is wise to use high-dose corticosteroids in the acute faze of the choroiditis, when we speak about long-term treatment, we think at immuno-modulatory agents [**23**].

In our patient, we used high dose prednisone (dose of 1 mg per kg) for 36 hours, and then we tapered the dose for a period of 10 weeks. Corticosteroids may prevent the apparition of the recurrences, but thy have to be given in high doses, and also for a prolonged period. In this case, we must pay attention to the significant complication of long-term use of the corticosteroid. 

Other medication available includes immunomodulatory agents – cyclosporine (with long term control of the inflammation and a low complication rate [**22**]; antimetabolites – azathioprine, mycophenolate mofetil; alkylating agents – chlorambucil and cyclophosphamide; biological agents – interferon alpha; anti-tumor necrosis factor (TNF) agents.

We also have options for local treatment, which are topical administration for the corticosteroid, in the situation of an anterior segment inflammation [**20**], or other kind of drug administration (intravenous, peribulbar, subtenon, and intravitreal). When systemic corticosteroids are contraindicated, intravitreal injection with triamcinolone acetonide is available [**19**]. In our patient, we also used dexamethasone in topical administration for 5 times per day initially and tapered for 4 weeks. 

The visual acuity has improved from 0.5 to 1 within 12 weeks. Long-term prognosis in this case of disease is guarded, with a range from a visual acuity of no light perception to a maximum of 1, with a natural course for progressive choroiditis, which involves recurrences and also involvement of new areas of retina [**24**]. Close monitoring when we taper the corticosteroid is a mandatory status for the preservation of visual function. 

## Conclusions

SC is an inflammation, often bilateral, also recurring and asymmetric progressive situated at the level of the inner choroid. The disease affects the choriocapillaris, but also the RPE. Till now, the process seems to be primarily idiopathic, but are theories that proves an autoimmune process. Regarding the fundus auto-fluorescence, the imagistic investigation is improving the diagnostic accuracy, and also the follow-up and management, when we think of the determination in the visual prognosis. Local treatment is an effective treatment, mostly for the SC’s complications. 

**Acknowledgements**

All authors have equal contribution.
